
JOURNAL INFORMATION
==============================
NLM Title Abbreviation: ScientificWorldJournal
Journal ID: ScientificWorldJournal
NLM Journal ID: 101131163
Journal ID: TSWJ

The Scientific World Journal

ISSN: 2356-6140
EISSN: 1537-744X
Publisher: Wiley

ARTICLE INFORMATION
==============================
PMCID: PMC6009748
PMID: 29973821
DOI: 10.1100/tsw.2002.47
Article ID: 571026
Article version: 1
Subjects: Short Report

IA3, A Yeast Proteinase A Inhibitor, Is Intrinsically Unstructured in Solution

Green Terry 1
Perry Kyle 1
Smith Leif 1
Phylip Lowri H. 2
Logan Timothy M. 3
Hagen Stephen J. 4
Dunn Ben M. 1
Edison Arthur 1 *
Departments of Biochemistry & Molecular Biology, University of Florida, Gainesville, FL 32610-0245, USA
School of Biosciences, Cardiff University, P. O. Box 911, Cardiff CF10 3US, Wales, USA
Department of Chemistry and Program in Molecular Biophysics, Florida State University, Tallahassee, FL 32306, USA
Physics, Box 100245, University of Florida, Gainesville, FL 32610-0245, USA
*Arthur Edison: art@ascaris.ufbi.ufl.edu
Publication date: 2002
Electronic publication date: 2002 May 15
Volume: 2
First page: 99
Last page: 101
Copyright: Copyright © 2002 Terry Green et al.
Copyright year: 2002
License: This is an open access article distributed under the Creative Commons Attribution License, which permits unrestricted use, distribution, and reproduction in any medium, provided the original work is properly cited.
License URL: https://creativecommons.org/licenses/by/3.0/

Funding: University of Florida National High Magnetic Field Laboratory Biotechnology and Biological Sciences Research Council 72/C13544 National Institutes of Health 1R01AI49063-01

==============================
Advances in Gene Technology: The Genome and Beyond –
Structural Biology for Medicine (Proceedings of the 2002 Miami
*Nature Biotechnology* Winter Symposium)
*TheScientificWorld* 2002, 2(S2), 99–101
ISSN 1532-2246; DOI 10.1100/tsw.2002.47

### $IA_3$, A YEAST PROTEINASE A INHIBITOR, IS INTRINSICALLY UNSTRUCTURED IN SOLUTION

Terry Green[1], Kyle Perry[1], Leif Smith[1], Lowri H. Phylip[2], Timothy M. Logan[3], Stephen J. Hagen[4], Ben M. Dunn[1], and Arthur S. Edison[1,*]
[1]Departments of Biochemistry & Molecular Biology and [4]Physics, Box 100245, University of Florida, Gainesville, FL 32610-0245; [2]School of Biosciences, Cardiff University, P. O. Box 911, Cardiff CF10 3US, Wales; [3]Department of Chemistry and Program in Molecular Biophysics, Florida State University, Tallahassee, FL 32306
*art@ascaris.ufbi.ufl.edu

**INTRODUCTION.** $IA_3$ is a 68 amino acid yeast protein that inhibits yeast proteinase A (YprA) with a $K_i$ of about 1 nM. No other aspartic proteinase has been found to be inhibited by $IA_3$, and at least 15 aspartic proteinases related to YprA cleave $IA_3$ as a substrate[1,2]. An X-RAY crystal structure has shown that the first 34 amino acids of $IA_3$ binds to YprA as an $\alpha$-helix[3]. $IA_3$ does not crystallize in the absence of YprA, and we have now found by NMR and CD that $IA_3$ is unfolded in solution and undergoes a large structural transition upon binding to YprA.

**METHODS.** Recombinant $IA_3$ with a His-tag for purification was produced in *E. coli* with $^{15}N$ or $^{15}N/^{13}C$ uniform isotopic enrichment. Triple resonance NMR was used to assign most of the backbone and side-chain resonances of free $^{15}N/^{13}C$–labeled $IA_3$. Samples of $^{15}N$-labeled $IA_3$ were then added to natural abundance YprA and were titrated with YprA and TFE (trifluoroethanol). Circular dichroism (CD) was also used to monitor changes of $IA_3$ upon addition of TFE. The inhibition constants ($K_i$) of $IA_3$ for YprA were determined as a function of temperature for van't Hoff analysis.

**RESULTS.** The chemical shifts of free $IA_3$ were found to be essentially random-coil values. The CD spectrum of $IA_3$ was characteristic of a random-coil peptide but changed to $\alpha$-helix upon addition of approximately 30% TFE. The $^{15}N$-HSQC NMR spectra of $IA_3$ were characteristic of random-coil structure, but the addition of YprA produced a large structural transition in $IA_3$ (Fig. 1). The $IA_3$ samples used for NMR analysis were active and inhibited YprA with a $K_i$ of about 2 nM. $^1H$-$^{15}N$ heteronuclear NOEs at 600 MHz of free $IA_3$ were very small or negative, consistent with the expected dynamics of an unfolded protein. A van't Hoff analysis of the temperature-dependence of the Ki of $IA_3$ for YprA was nonlinear, suggesting a rather complex coupled equilibria of protein folding and inhibition (Fig. 2).

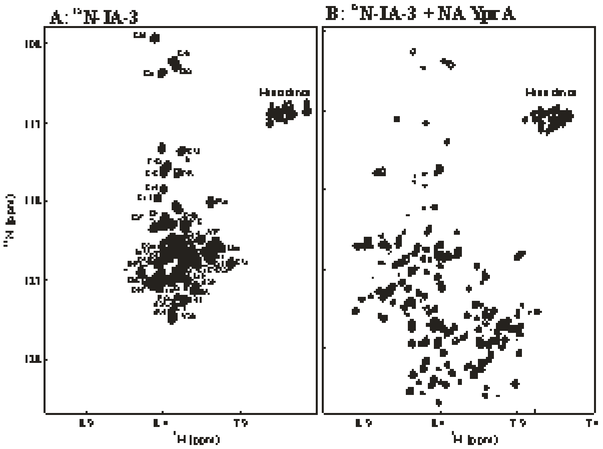

**FIGURE 1**. $^{15}$N-HSQC of free $^{15}$N-labeled $IA_3$ (left) and $^{15}$N-labeled $IA_3$ and unlabed YprA (right). The spectrum on the right was obtained from the sample shown on the left by adding an approximately stoichiometric amount of unpurified YprA (Sigma).

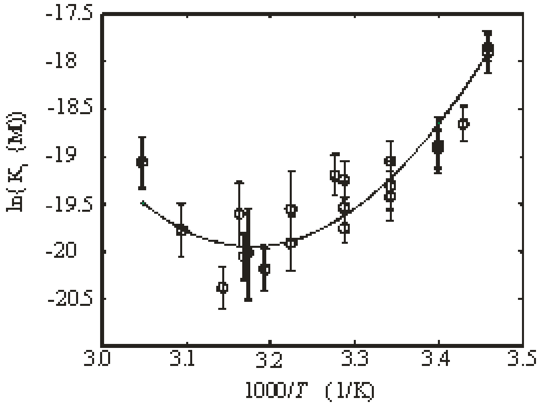

**FIGURE 2.** van't Hoff plot of the inhibition of YprA by $IA_3$. A standard 2-state thermodynamic analysis of the data generates the fit shown, which indicates a positive van't Hoff enthalpy $\Delta H(T_0) = H_{bound}(T_0) - H_{unbound}(T_0) = 76 \pm 9$ kJ/mol and $\Delta Cp = -4.6 \pm 1.2$ kJ/mol-K, at a reference temperature $T_0 = 25°C$. These data and the simple model therefore suggest that $IA_3$ binds endothermically to YprA at room temperature, but with an overall heat capacity change that decreases with temperature, so that binding becomes exothermic near 40°C. The entropy of binding is not well determined in this type of analysis. However, this standard two-state thermodynamic model may be regarded as oversimplifying the actual binding, which entails both protein folding and ligand binding.

**DISCUSSION.** $IA_3$ has a large structural transition upon binding to YprA. NMR and CD data have shown that the fully active $IA_3$ is unstructured in solution, and X-RAY data have shown that the N-terminal half of $IA_3$ forms a near-perfect $\alpha$-helix in complex with YprA. The precise mechanism of inhibition of YprA by $IA_3$ is still unclear. Specifically, we do not yet know whether YprA selects very small (currently undetectable) amounts of $\alpha$-helical $IA_3$ for binding or whether YprA provides a template upon which $IA_3$ folds into a helix. More thermodynamic measurements are needed, but the van't Hoff analysis suggests that the folding and inhibition steps are coupled together. To sort out the steps of folding and binding, we are currently designing major modifications to $IA_3$ to force it to become an $\alpha$-helix in solution.

**ACKNOWLEDGMENT.** This work was supported by the University of Florida College of Medicine, the National High Magnetic Field Laboratory, the BBSRC (72/C13544 to LHP) and the NIH (1R01AI49063-01 to BMD). NMR data were collected at the Advanced Magnetic Resonance Imaging and Spectroscopy Facility at the McKnight Brain Institute of the University of Florida.

**REFERENCES**

1. Dreyer, T., Valler, M.J., Kay, J., Charlton, P., and Dunn, B.M. (1985) Biochem. J. 231, 777–779.
2 Phylip, L.H., Lees, W.E., Brownsey, B.G., Bur, D., Dunn, B.M., Winther, J.R., Gustchina, A., Li, M., Copeland, T., Wlodawer, A., and Kay, J. (2001) J. Biol. Chem. 276, 2023–2030.
3. Li, M., Phylip, L.H., Lees, W.E., Winther, J.R., Dunn, B.M., Wlodawer, A., Kay, J., and Gustchina, A. (2000) Nat. Struct. Biol. 7, 113–117.
